# The Effect of a Combined Intervention Program Combining Cognitive Training and Physical Activity in Community‐Dwelling Elderly Without Cognitive Impairment

**DOI:** 10.1002/alz.090203

**Published:** 2025-01-09

**Authors:** Ye‐Eun Shin, Myeong‐Il Han, Kyung‐Hwan Park, Mal‐Rye Choi, Nam‐Ju Sung, Sol‐Yi Kim

**Affiliations:** ^1^ Presbyterian Medical Center, Jeonju Korea, Republic of (South); ^2^ Jeonbuk Provincial Dementia Center, Jeonju Korea, Republic of (South)

## Abstract

**Background:**

Combined cognitive training and physical activity has been known to improve brain function. The purpose of this study is to investigate whether combined intervention affects the improvement of cognitive function in the community‐dwelling elderly, and to determine if it improves physical function, such as motor speed and balance.

**Method:**

The study was conducted among community‐dwelling elderly aged 65 years. Participants had no evident cognitive impairment on the Cognitive Impairment Screening Test (CIST), and be physically active. Demographic data was collected, and cognitive function was assessed using a neuropsychological test battery which consists of the Word List Test, Organization Skills, TMT, Word Recall, Boston Naming Test, and orientation. Physical functioning was assessed using the Brief Physical Performance Battery (SPPB), which consists of balance, gait speed, and chair rise assessments. The intervention was a group‐based, combines cognitive training and physical activity and allows to perform simultaneously, with a total of eight sessions, each lasting 60 minutes. Differences were tested between pre‐intervention and post‐intervention scores.

**Result:**

A total of 73 participants completed the intervention and were eligible for analysis. After the intervention training, the total score of the neurocognitive test battery increased by 7.32 (±4.20) points (p<0.001) and the SPPB increased by 1.59 (±1.22) points (p<0.001), respectively. In the detailed items of the neurocognitive test battery, word memory was higher in men 3.71 than in women 2.05 (p = .013), and word recall was higher in women 1.44 than in men 0.36 (p = .013), which both reveal significant difference. According to age, orientation was significantly different with a score of 1.45 for those aged 85 years and older than 0.46 for those aged 75‐79 years (p = .044). There were no significant differences in BMI and living arrangements. Multiple regression analysis showed that education (secondary or higher) (p = .008), living arrangement (children) (p = .042), hypertension medication (p = .014), and diabetes medication (p = .015) had a significant effect on neurocognitive performance of this intervention.

**Conclusion:**

The results demonstrated that combined intervention improved overall cognitive function and physical performance in community‐dwelling elderly with no apparent cognitive impairment.

